# Immunosuppressants exert antiviral effects against influenza A(H1N1)pdm09 virus via inhibition of nucleic acid synthesis, mRNA splicing, and protein stability

**DOI:** 10.1080/21505594.2023.2301242

**Published:** 2024-01-03

**Authors:** Xin Wang, Feiyang Pu, Xuanye Yang, Xili Feng, Jiayou Zhang, Kai Duan, Xuanxuan Nian, Zhongren Ma, Xiao-Xia Ma, Xiao-Ming Yang

**Affiliations:** a Key Laboratory of Biotechnology and Bioengineering of State Ethnic Affairs Commission, Biomedical Research Center, Northwest Minzu University, Lanzhou, China; b School of Stomatology, Lanzhou University, Lanzhou, China; c National Engineering Technology Research Center for Combined Vaccines, Wuhan, China; d Wuhan Institute of Biological Products Co, Ltd, Wuhan, China; e China National Biotech Group Company Limited, Beijing, China

**Keywords:** Influenza A virus, immunosuppression, Immunosuppressants, antiviral agents

## Abstract

Influenza A virus (IAV) poses a threat to patients receiving immunosuppressive medications since they are more susceptible to infection with severe symptoms, and even death. Understanding the direct effects of immunosuppressants on IAV infection is critical for optimizing immunosuppression in these patients who are infected or at risk of influenza virus infection. We profiled the effects of 10 immunosuppressants, explored the antiviral mechanisms of immunosuppressants, and demonstrated the combined effects of immunosuppressants with the antiviral drug oseltamivir in IAV-infected cell models. We found that mycophenolic acid (MPA) strongly inhibits viral RNA replication via depleting cellular guanosine pool. Treatment with 6-Thioguanine (6-TG) promoted viral protein degradation through a proteasomal pathway. Filgotinib blocked mRNA splicing of matrix protein 2, resulting in decreased viral particle assembly. Furthermore, combined treatment with immunosuppressants and oseltamivir inhibits IAV viral particle production in an additive or synergic manner. Our results suggest that MPA, 6-TG, and filgotinib could be the preferential choices for patients who must take immunosuppressants but are at risk of influenza virus infection.

## Introduction

Influenza A viruses (IAVs) account for most of the seasonal epidemics and all known flu pandemics. Around 5–10% adults and 20–30% children were attacked, and about 500,000 deaths were caused annually by influenza infection [1]. Influenza viruses, belonging to the *Orthomyxoviridae* family and classified into four types, are enveloped viruses containing a single-stranded RNA genome with eight viral RNA segments encoding 10 viral proteins [2]. The envelope of IAV is decorated by integral membrane proteins, hemagglutinin (HA), neuraminidase (NA), and matrix protein 2 (M2). Both glycoproteins HA and NA are responsible for the entry and release stage of IAV life cycle and their synthesis requires the addition of the glycan moiety in the endoplasmic reticulum (ER), resulting in disruption of ER homeostasis and activation of unfolded protein response (UPR) [3–5]. Ion channel protein M2 shares the RNA segment with matrix protein 1 (M1) and IAV nonstructural-protein 1 (NS1) interacts with host protein to splice *M1* mRNA with host splicing machinery [6,7]. Despite efforts on drugs and vaccines, higher incidence rate and significant morbidity and mortality from IAV infection are associated with immunosuppressed population [8].

Organ transplant recipients and individuals with autoimmune diseases or inflammatory bowel diseases necessitate the administration of immunosuppressive medications to mitigate immune system-mediated assaults on transplanted organs and healthy host cells. Administration of immunosuppressants suppresses alloimmunity and autoimmunity, and at the same time, compromises the host in combating viral infections. Infectious diseases highly threaten this population [8]. Of all the vaccine-preventable infections, influenza has the highest incidence rate in immunocompromised population [8]. However, the impact of immunosuppressants on influenza infection can be multifaceted. The severity of influenza infection is determined by the interplay between the viral virulence and the host immune response. The cytokine storm, one of the reasons for poor clinical outcome, triggered by extension of virus infection to the lung, causes serious complications, including acute respiratory distress syndrome and multiple-organ dysfunction syndrome [9,10]. To weaken the cytokine storm, various immunosuppressants have been advised as the treatment [11]. Concern may arise that immunosuppressive agents promote viral replication and interact with anti-flu drugs, such as oseltamivir.

Given the substantial clinical burden of severe influenza in immunocompromised population, it is essential to determine the direct effects of immunosuppressants on influenza virus infection and to understand their interactions with oseltamivir. Therefore, we profiled the effects of different clinically used immunosuppressants on IAV in cell culture models, including corticosteroids (dexamethasone, prednisone), calcineurin inhibitors (cyclosporine, tacrolimus), mammalian target of rapamycin (m-TOR) inhibitors (rapamycin, everolimus), purine and pyrimidine synthesis inhibitors (mycophenolic acid [MPA], 6-thioguanine [6-TG]), and JAK inhibitors (filgotinib, tofacitinib). We found three drugs exerting anti-flu effects through different mechanisms, MPA inhibiting the synthesis of guanosine monophosphate, filgotinib blocking M2 splicing and 6-TG promoting HA degradation. Furthermore, we demonstrated the effects of these three drugs when combined with oseltamivir. These results provide a reference for balancing tackling alloimmunity and autoimmunity and combating influenza infection, and immunosuppressant-oseltamivir combined treatment for severe influenza.

## Materials and methods

### Cells and viruses

Human lung adenocarcinoma A549 cells (ATCC #CCL-185) and Madin-Darby canine kidney (MDCK) cells (ATCC #CCL-34) were provided by Biomedical Research Center, Northwest Minzu University, Lanzhou, China, and maintained in Dulbecco’s modified Eagle’s medium (DMEM) (Lanzhou Minhai Bio-engineering, China) supplemented with 10% fetal bovine serum (FBS) (Lanzhou Minhai Bio-engineering, China) at 37°C under 5% CO_2_ atmosphere.

Influenza virus A/Singapore/GP1908/2015 (IVR-180) was provided by National Engineering Technology Research Center for Combined Vaccines, Wuhan, China, and were propagated in 10-day-old embryonated chicken eggs at 37°C for 72 h.

### Reagents and antibodies

Dexamethasone (CAS no.50-02-2), prednisone (CAS no.53-03-2), cyclosporine (CAS no.79217-60-0), tacrolimus (CAS no.104987-11-3), rapamycin (CAS no.53123-88-9), everolimus (CAS no.159351-69-6), MPA (CAS no.24280-93-1), 6-TG (CAS no.154-42-7), and MG-132 (CAS no.133407-82-6) were purchased from Aladdin (Shanghai, China). Filgotinib (CAS no.1206161-97-8), tofacitinib (CAS no.477600-75-2), oseltamivir acid (CAS no.187227-45-8) were purchased from MCE China. Oseltamivir acid was dissolved in ddH_2_O, and other drugs were dissolved in DMSO.

Antibodies targeting IAV NP (Rabbit, GTX636247), IAV H1N1 HA (Rabbit, GTX127357), M1 (Rabbit, GTX125928), M2 (Rabbit, GTX125951), and NS1 (Rabbit, GTX125990) were purchased from GeneTex (Shanghai, China). The GAPDH antibody (AP0066) was purchased from Bioworld (Nanjing, China). HRP-conjugated anti-rabbit IgG (A21020) was purchased from Abbkine (Wuhan, China).

### Drug screen

For virus infection, A549 cells were infected with influenza A virus diluted by DMEM at an MOI of 0.1 for 2 h at 37°C. Inoculum was then removed, cells were washed twice with phosphate-buffered saline (PBS) and added with infection medium (DMEM supplemented with 2 μg/mL TPCK-treated trypsin) with drugs or DMSO. Cell samples were collected for quantitative Real-Time PCR and western blotting, and cell culture supernatant were collected for plaque assay and hemagglutination assay.

### RNA extraction and quantitative real-time PCR

TRIzol reagent (Biosharp, China) was used to extract total RNA from cell samples, and total RNA was then reverse transcribed into complementary DNA (cDNA) with the reverse transcription system from Vazyme (Nanjing, China). The quantitative Real-Time PCR was performed with SYBR Green (Vazyme, Nanjing, China) following the instructions, and the reaction system was as follows: cDNA: 1 μL, forward primer (10 μM): 0.5 μL, reverse primer (10 μM): 0.5 μL, qPCR Master Mix: 10 μL, RNase Free Water: 8 μL. The relative amount of each gene was normalized to the amount of GAPDH, and the relative expression of the target genes was calculated by 2^−ΔΔCT^. To quantify the IAV RNA replication, IAV PB1 was probed with PB1 forward primer (F): 5’-ATGGAATATGACGCTGTTG-3’ and reverse primer (R): 5’-TTGGCTTGTGTTGAGAATAG-3.’ Sequence of primers specific for GAPDH was as follows: F: 5’-CTCTGGTAAAGTGGATATTGT-3’ and R:5’-GGTGGAATCATATTGGAACA-3.’ To quantify the relative mRNA level of M1 and M2, the used primers were listed: M1 F: 5’-TGGAGGTTGCTAATAAGA-3,’ R: 5’-CTGCAAATTTTCAAGAAGGTCATCTCT-3’; M2 F: 5’-GTCGAAACGCCTACCAGAAG-3,’ R: 5’-CTCTAGCTCTATGTTGACAAAATGACC-3.’

### Western blotting

Cell samples were collected, and total protein was extracted with RIPA. The total protein concentration was determined by BCA method (Biosharp, China). Fifteen μg protein sample was loaded on to 8% or 15% SDS-PAGE and transferred to polyvinylidene difluoride (PVDF) membranes (pore size, 0.2 μm; Sigma-Aldrich, USA). After transfer, membranes were blocked for 1 h at ambient temperature and then incubated with primary antibodies overnight at 4°C plus secondary antibodies for 1 h at ambient temperature. Proteins were detected by an electrochemiluminescence detection system. The images were analyzed with ImageJ 1.8 software.

### Plaque assay

A confluent monolayer of MDCK cells was infected with diluted cell culture supernatant for 2 h. Then, the inoculum was removed and an agarose overlay, containing 1X DMEM, 1% agarose (CAS no.9012-36-6; Sigma-Aldrich, USA) and 2 μg/mL TPCK-treated trypsin, was added. After incubation at 37°C for 72 h, cells were fixed with 4% paraformaldehyde for 1 h and stained with crystal violet.

### Hemagglutination assay

The cell culture supernatant was serially diluted with 0.9% saline solution, and 25 μL of the diluted supernatant was added into a U-bottom shaped 96-well microtiter plate. Twenty-five μL of chicken erythrocytes (1% v/v in PBS) was added to each well and gently mixed. The mixture was incubated at 4°C for 30 min to allow hemagglutination.

### Synergy analysis

To evaluate the combined effects of oseltamivir and immunosuppressants on IAV replication, the Bliss method in SynergyFinder 2.0 [12] was adopted to analyze the data of 36 h combined treatment of oseltamivir and immunosuppressants on IAV-infected A549 cells. When the combination is additive, the synergy score lies between −10 and 10. A synergy score higher than 10 indicates a synergistic effect of the combination, and a synergy score lower than −10 indicates antagonism. The 95% confidence interval was considered to be statistically significant.

### Statistical analysis

All numerical results are presented as mean ± standard error of the mean (SEM). Comparisons between the groups were assessed with ANOVA test using GraphPad Prism 9 (GraphPad Software, Inc., La Jolla, CA, USA). Significance is indicated in the figures (*, *p* value of < 0.05; **, *p* value of < 0.01; ***, *p* value of < 0.001).

## Results

### Most immunosuppressants have no antiviral effects on IAV replication

**Figure 1.**
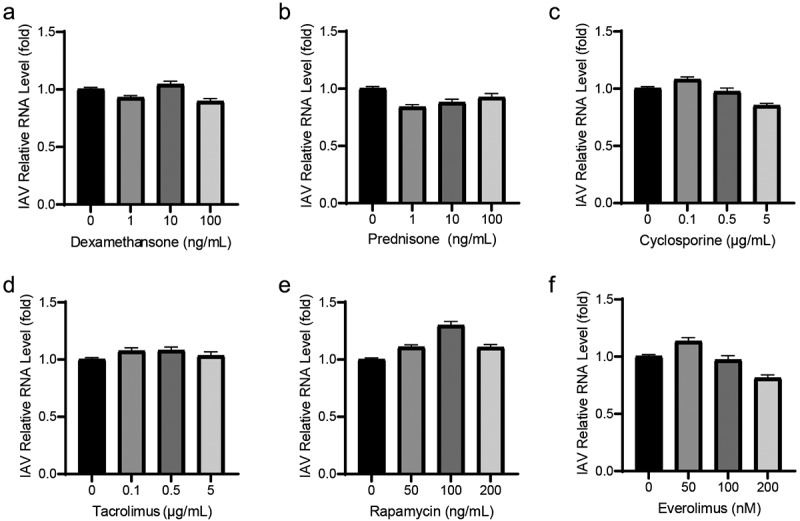
Most immunosuppressants have no antiviral effects on IAV replication. Corticosteroids, including dexamethasone (a) and prednisone (b) showed no antiviral effects on IAV RNA replication. Cyclosporine (c) and tacrolimus (d) also had no antiviral activities. Similarly, no inhibition on IAV RNA replication were detected on rapamycin (e) and everolimus (e). A549 cells were infected with A/Singapore/GP1908/2015 (H1N1) at MOI of 0.1 and were treated with immunosuppressants for 36 h. IAV RNA level was quantified by qRT-PCR and compared to control group treated with 1‰ DMSO (36 h, *n* = 3 independent experiments with two or three replicates each). Data are presented as the means ± SEM.

To examine the effects of immunosuppressants on IAV replication, human alveolar epithelial cells (A549) were infected with A/Singapore/GP1908/2015 (H1N1) and treated with these compounds for 36 h. Then, the yields for viral RNA copies were determined by qRT-PCR. Corticosteroids (dexamethasone and prednisone), which are the first generation of immunosuppressants, were evaluated, and they had no significant antiviral effect on IAV replication (Figure 1a,b). Calcineurin inhibitors (cyclosporine, tacrolimus) (Figure 1c,d) and m-TOR inhibitors (rapamycin, everolimus) (Figure 1e,f) also had no clear effect on IAV replication.

### MPA potently inhibits IAV replication through depletion of the cellular guanosine nucleotide pool

**Figure 2.**
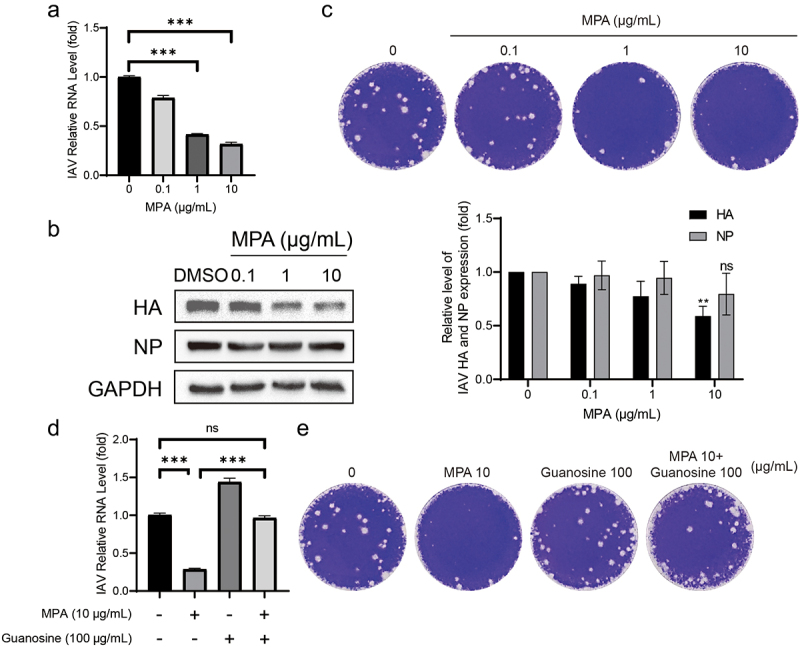
MPA potently inhibits the multiplication of IAV through depletion of the cellular guanosine nucleotide pool. (a) MPA significantly inhibited IAV RNA replication. A549 cells were infected with A/Singapore/GP1908/2015 (H1N1) and treated with MPA for 36 h. IAV RNA level were quantified by qRT-PCR and compared to 1‰ DMSO treated group (*n* = 3 independent experiments with two or three replicates each). (b) IAV HA accumulation was inhibited by MPA. Lysates of IAV-infected A549 cells were subjected to western blotting for IAV HA and NP. GAPDH was used as loading control. Images are representative of three independent experiments. (c) MPA reduced virion production. Supernatant from MPA treatment (36 h) on IAV-infected A549 cells were analysed by plaque assay. MDCK cells were infected with diluted supernatant and after 72hpi, cells were fixed by formaldehyde and stained with crystal violet. Images are representative of three independent experiments with six replicates each. Exogenous guanosine was supplemented to MPA treatment on IAV- infected A549 cells, and IAV RNA level (d) and viral particle production (e) were analysed by qRT-PCR and plaque assay, respectively. Data are presented as the means ± SEM (*, *p* < 0.05; **, *p* < 0.01; ***, *p* < 0.001).

The main purine synthesis inhibitors used in the clinic are azathioprine and MPA. Compared to azathioprine, MPA and its prodrugs are more widely used. Previous researches have proven that MPA has significant antiviral effects on various viruses including dengue virus and MERS [13–15]. MPA treatment of IAV infected A549 cells with 0.1, 1, 10 μg/mL displayed a significant inhibition of IAV RNA replication in a dose-dependent way (Figure 2a). The reduction induced by MPA ranged from 21 ± 3% to 68 ± 2% (*n* = 6; *p* < 0.05) as the concentration of MPA rose. Despite no significant inhibition on NP synthesis, MPA can inhibit the synthesis of viral protein HA by 41 ± 5% (*n* = 4; *p* < 0.05) (Figure 2a,b). Furthermore, plaque assay was performed to detect the yield of viral particles in the cell culture supernatant. MPA can inhibit the virion release at a concentration of 1 and 10 μg/mL (Figure 2c).

MPA, as an antimetabolite drug, disrupts cell proliferation via inhibiting inosine monophosphate dehydrogenase (IMPDH) and as a result impairs the formation of guanosine monophosphate (GMP) in *de novo* synthesis. Without sufficient GMP, viral RNA replication will be blocked. Nucleotide salvage pathways can use guanosine to synthesize GMP. To validate this hypothesis, exogenous guanosine was added. Supplemented with an exogenous guanosine (100 μg/ml), the yields of RNA copies and of viral particles increased obviously (Figure 2d,e). Supplementation of guanosine significantly abolished the antiviral effects of MPA. This confirmed that MPA depleted the cellular GMP pool and inhibited the IAV viral RNA replication.

### 6-TG inhibits IAV replication via promoting HA proteolytic degradation

**Figure 3.**
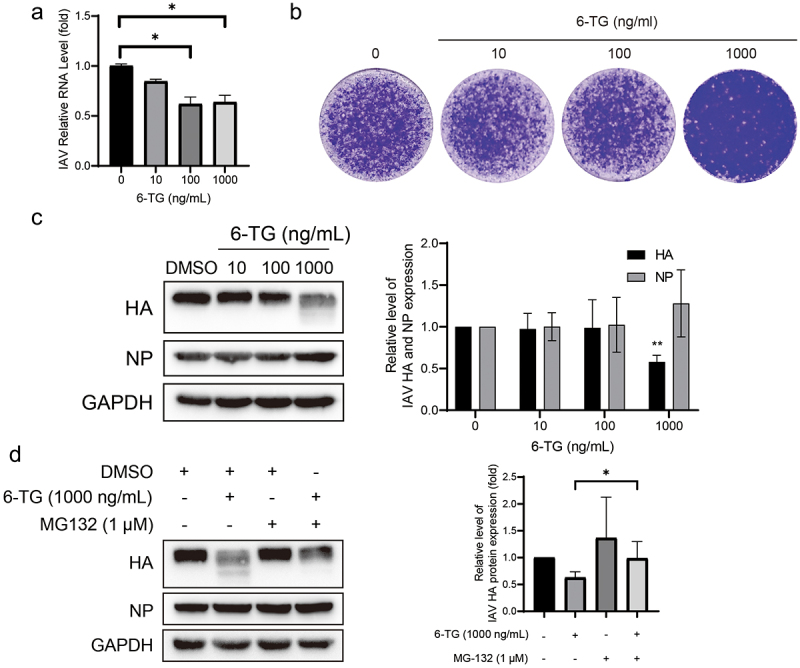
6-TG inhibits IAV replication via promoting HA proteolytic degradation. (a) 6-TG moderately inhibited IAV RNA replication. IAV-infected A549 cells were treated with 6-TG for 36 h and viral RNA were quantified by qRT-PCR, normalized to GAPDH (*n* = 3 independent experiments with two or three replicates each). (b) 6-TG reduced IAV HA accumulation at 1000 ng/mL dose. Western blotting analysis of IAV HA and NP. Images are representative of three independent experiments. (c) 6-TG significantly reduced virion production at 1000 ng/mL dose. Viral particle production was analysed by plaque assay. Images are representative of three independent experiments with six replicates each. (d) 6-TG-induced HA degradation was blocked by MG132. Together with 6-TG (36 h), MG132 (1 μM) was add for 36 h to block proteasome-dependent protein degradation. Cell lysates were subjected to western blotting for IAV HA protein level. Images are representative of three independent experiments. Data are presented as the means ± SEM (*, *p* < 0.05; **, *p* < 0.01; ***, *p* < 0.001).

Azathioprine is a purine synthesis inhibitor, and 6-Thioguanine (6-TG) is the active metabolite of azathioprine. As shown in (Figure 3a), 6-TG inhibited viral RNA replication to some degree, but its performance was weaker than that of MPA. 6-TG reduced IAV RNA by 36 ± 7% (*n* = 6; *p* < 0.05) at a concentration of 1000 ng/mL. Of note, a sharp decrease in viral particle production was observed, and this phenomenon represented a dose-dependent manner (Figure 3b). In addition, significant inhibition of viral protein can be observed in 6-TG treatment. 6-TG imposed no effect on inhibiting HA accumulation at concentrations of 10 and 100 ng/mL, while the 39 ± 5% reduction of HA accumulation (*n* = 3; *p* < 0.05) was observed at the concentration of 1000 ng/mL, except for NP production (Figure 3c).

Since smearing can be observed below the dominant band, we hypothesized that the reduced accumulation of HA probably resulted from viral protein degradation rather than inhibition of synthesis. Moreover, proteasomes might take part in HA degradation in UPR. Subsequently, MG132, a proteasome inhibitor, was additionally treated. It has been observed that MG-132 can prevent protein degradation induced by 6-TG treatment (Figure 3d), suggesting that 6-TG induced HA proteolytic degradation.

### Filgotinib impairs IAV replication via suppressing M1 mRNA splicing

**Figure 4.**
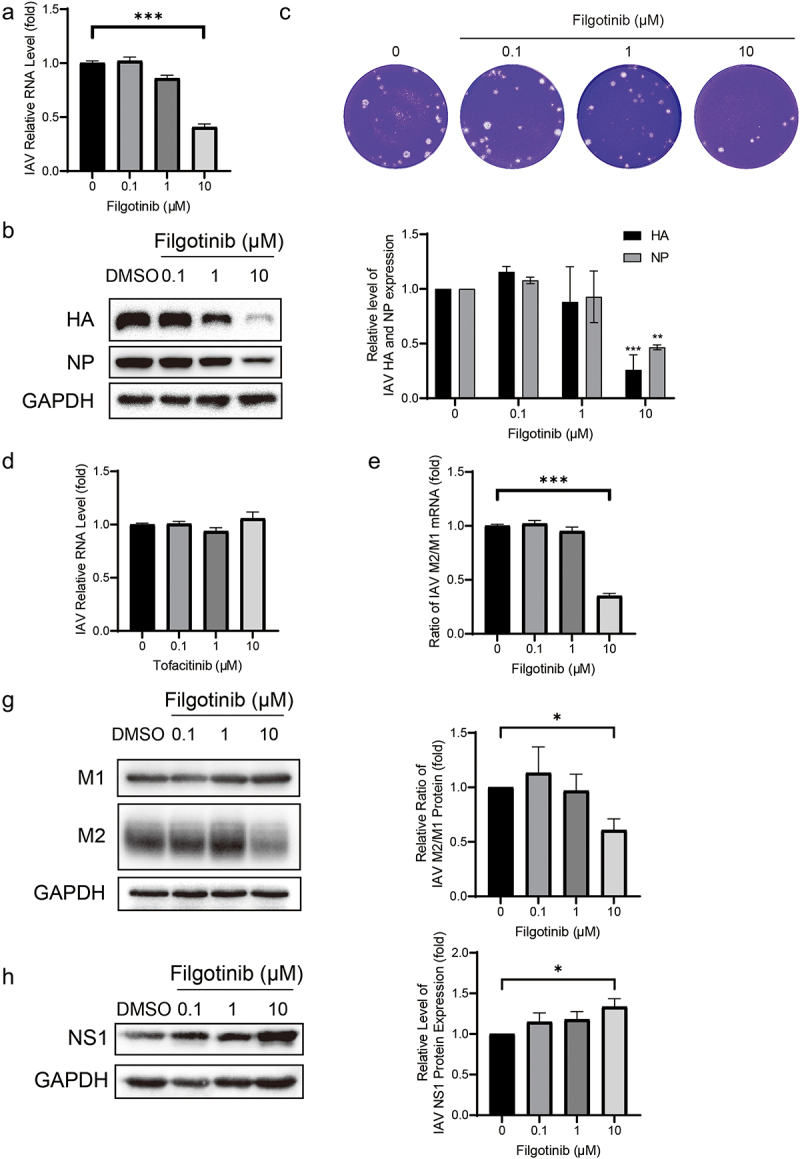
Filgotinib reduces IAV replication through suppressing M2 splicing. (a&d) Filgotinib significantly reduced IAV RNA level. Viral RNA was quantified by qRT-PCR after 36 h filgotinib and tofacitinib treatment and compared to control group (*n* = 3 independent experiments with two or three replicates each). (b) Filgotinib inhibited IAV HA and NP accumulation. Samples were subjected to western blot and HA and NP were detected. Experiments were replicated three times. (c) Filgotinib moderately reduced IAV virion production. Cell culture supernatant were analysed by plaque assay. images are representative of three independent experiments. (e) Filgotinib blocks M2 splicing. IAV M2 and M1 mRNA were quantified by qRT-PCR after 36 h filgotinib treatment. (g) M2 and M1 protein levels were detected by western blot and expression level were analysed. (h) NS1 protein levels were detected by western blot and expression level were analysed. Data are presented as the means ± SEM (*, *p* < 0.05; **, *p* < 0.01; ***, *p* < 0.001).

Filgotinib, a JAK1 inhibitor, was recently approved by the FDA to treat inflammatory bowel disease. In the IAV-infected cell model, 10 μM filgotinib significantly inhibited the IAV RNA level by 59 ± 3% (*n* = 7; *p* < 0.05; Figure 4a). Similar to effects on RNA, the yields of viral proteins (HA and NA) were significantly reduced by 10 μM filgotinib treatment (Figure 4b). HA and NA accumulation were reduced by 74 ± 14% and 53 ± 12% (*n* = 3; *p* < 0.05), respectively (Figure 4b). Consistently, the viral particle yield was also decreased in a dose-dependent manner (Figure 4c).

To further investigate the relationship between antiviral activity and JAK inhibition, tofacitinib, another JAK inhibitor, was employed to treat IAV-infected cells at 0.1, 1, and 10 μM doses. No antiviral effects were detected by tofacitinib treatment (Figure 4d). These results highlight that the underlying mechanism of filgotinib against IAV infection may be independent of JAK inhibition.

Influenza viruses hijack host splice factors to splice viral M1 mRNA to generate M2 mRNA [16]. Filgotinib was previously reported to inhibit HIV-1 transcription by suppressing HIV-1 splicing and in the IAV strain we used, the M segment owned the same splicing signals as reported [16,17]. The splicing cases for M1 mRNA were further examined. As shown in (Figure 4e), the ratio of M2/M1 mRNA was reduced by 10 μM filgotinib by 65 ± 4% (*n* = 6; *p* < 0.05) and subsequently, M2 protein accumulation declined as well. The relative ratio of the M2/M1 protein expression declined by 39 ± 10% (*n* = 3; *p* < 0.05) (Figure 4g). NS1 has been reported to promote M1 mRNA splicing [7,18], and its protein level was further quantified. With the treatment of 10 μM filgotinib, NS1 protein level rose by 34 ± 10% (*n* = 6; *p* < 0.05) (Figure 4h), indicating filgotinib suppressed M1 mRNA splicing via an NS1-independent method. The results imply that filgotinib exerts its antiviral activity on IAV infection by influencing M1 mRNA splicing.

### Combinatory effects of immunosuppressants and anti-flu drug on IAV infection

**Figure 5.**
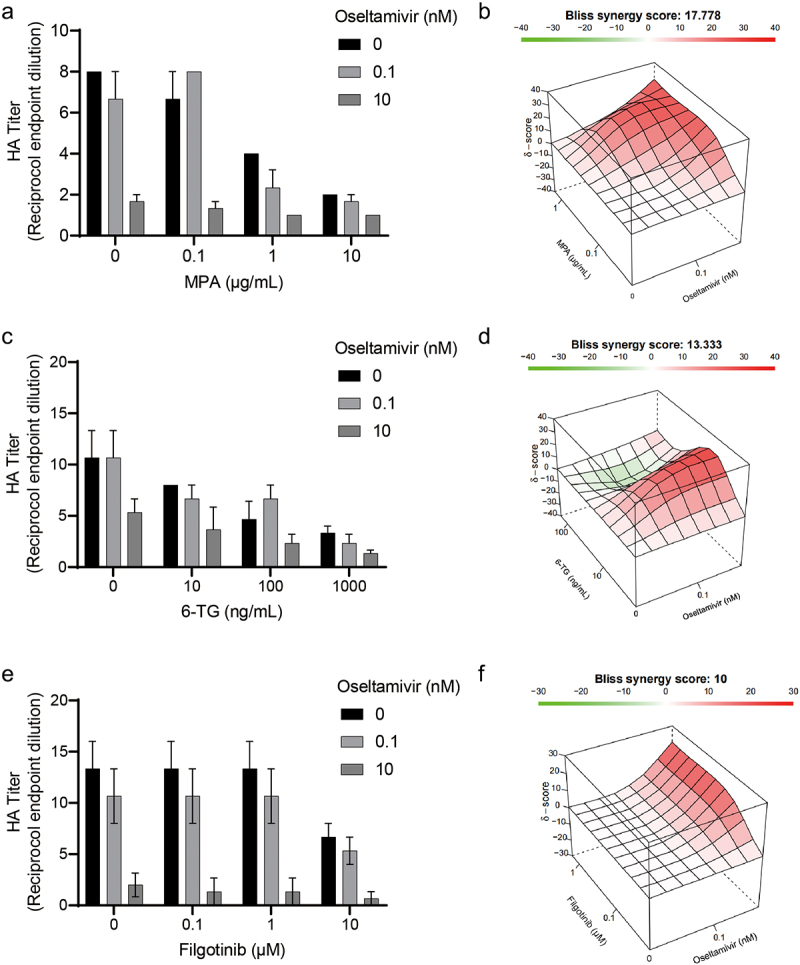
Combinatory effects of immunosuppressants and anti-flu drug on IAV replication. Oseltamivir at concentrations of 0.1 nM and 1 nM were used to treat IAV-infected A549 cells for 36 h together with MPA, 6-TG or filgotinib. The combinatory effect of MPA(a), 6-TG(c) or filgotinib(e) and oseltamivir were detected by haemagglutination assay and further analysed by SynergyFinder 2.0. The three-dimensional plots represent the synergy effects of MPA(b), 6-TG(d) or filgotinib(f) and oseltamivir.

Oseltamivir is widely used in preventing and treating influenza virus infection. Oseltamivir with 0.1 and 10 nM concentrations were, respectively, treated on IAV-infected cells together with MPA, 6-TG, or filgotinib to determine their combinatory effects on virion production (Figure 5a–e). These results were analyzed with SynergyFinder 2.0. All three selected immunosuppressive agents showed synergistic antiviral effects in combination with oseltamivir. Among all the combination groups, the combination of MPA and oseltamivir showed the strongest synergy, with a synergy score of 17.7 (Figure 5b). The synergy degree of filgotinib and oseltamivir was the lowest, as of 10 (Figure 5f), among the three combination groups.

## Discussion

The immune deficit resulted from the use of immunosuppressive agents in general facilitates viral infection. Infection caused by respiratory viruses remains a challenge to allograft function and survival and autoimmune disease management [8]. Although antiviral treatment and withdrawal of immunosuppression can reduce hospitalization and mortality [19], the prolonged viral shedding induced by impaired immunity and consequent viral drug resistance still pose an obstacle in managing respiratory viral infection [20]. Understanding the direct effects of immunosuppressants on influenza virus infection is of importance in this respect.

In this study, the IAV-infected A549 cells were used as a model to assess the effects of immunosuppressants on IAV infection. Ten FDA-approved immunosuppressants, classified into five categories, were tested for their potential antiviral effects. Most of these agents had no direct inhibitory activity on IAV replication, whereas MPA, 6-TG, and filgotinib exerted clear antiviral activities via different mechanisms.

The broad-spectrum antiviral activity of MPA has been reported for numerous viruses, including SARS-Cov-2, rotavirus, HCV, norovirus [21–23]. Its inhibition on IMPDH represents the main mechanism in restraining viral RNA replication and transcription [21,22,24]. We found that MPA treatment inhibited both IAV RNA level and protein production, resulting in reduced yield of viral particles (Figure 2a–c). Our result further expands the antiviral spectrum of MPA. Given that IMPDH plays a crucial role in the *de novo* synthesis of guanine nucleotides, essential for viral RNA replication, it is plausible that MPA could potentially demonstrate antiviral effects across a broad spectrum of RNA viruses. However, interpreting these cell model-based results into clinical practice remains challenging. A retrospective study showed that in liver transplant patients with COVID-19, the administration of mycophenolate mofetil (MMF), a prodrug of MPA, is associated with COVID-19 severity, although the mortality rate in this population is lower than that in the general population [25]. Thus, the direct antiviral effect of MPA in patients remains to be defined by well-designed clinical studies.

Similar to MPA, the immunosuppressive mechanism of 6-TG is also via inhibiting purine synthesis. Although we assumed that 6-TG might disrupt IAV RNA replication as a nucleoside analog, treatment of 6-TG affected the IAV RNA level only to a limited extent (Figure 3a). Previous research showed that 6-TG negatively regulated the replication of rotavirus and herpes simplex virus 1 by suppressing Rac1 but inhibition on Rac1 facilitated IAV replication [26–28]. Our results demonstrated that 6-TG promoted the degradation of HA (Figure 3d). Considering 6-TG is an anti-cancer chemotherapy drug and immunosuppressant, both cancer patients and transplantation recipients are vulnerable to influenza infection. It is inspiring to find that 6-TG has the ability to inhibit IAV replication.

The JAK-STAT pathway is activated by interferon receptors to produce hundreds of antiviral interferon-stimulated genes and proinflammatory cytokines [29]. JAK inhibition will disturb the interferon antiviral activity, but filgotinib significantly decreases viral RNA level and viral protein accumulation in IAV-infected cells (Figure 4a, b). The slight rise at 0.1 μM and 1 μM can be deemed as the result of JAK-STAT inhibition surpassing antiviral activity. Research on SARS-CoV-2 showed that expression of ACE2, the entry factor, was regulated by interferon, and JAK inhibitors can dampen the interferon-stimulated transcription of ACE2 [30,31]. However, few relationships between ACE2 and IAV infection have been established, and another JAK inhibitor, tofacitinib, had no antiviral effects (Figure 4d). Filgotinib fights against IAV infection via a JAK-independent pathway. A previous study pointed out that filgotinib played an antiviral role in HIV infection via blocking viral gene splicing [17]. Subsequently, we measured the yields of M2/M1 mRNA and the corresponding protein and found that filgotinib can disrupt the M1 mRNA splicing for impairing IAV infection via an unknown mechanism. Quantification of NS1 protein levels further suggests that the influenza A virus (IAV) may leverage the elevated levels of NS1 to counteract the antiviral effects induced by filgotinib.

Optimizing the clinical choices of immunosuppressants will help to better manage IAV infection, but anti-flu drugs, especially neuraminidase inhibitors, are the main force in combating IAV infection [20]. In this study, the combination of oseltamivir with different immunosuppressants represented positive antiviral synergy (Figure 5), supposing these antiviral immunosuppressive agents will not disturb the antiviral action of oseltamivir in immunosuppressed patients. Also, the combination results provide reference for treating severe influenza. Despite the effectiveness of oseltamivir in improving patients’ outcome, mortality in severe influenza remains a challenge and immunomodulatory therapy has been advised in suppressing the cytokine storm, an uncontrolled pro-inflammatory response that determines the outcome of severe influenza [11]. Due to their suppression on immune cells and multiple immune receptors, corticosteroids are regarded as a potent anti-inflammatory treatment, while several observational studies suggest that the use of corticosteroids in patients may not improve clinical outcomes and even cause hospital-acquired infections [32]. More drugs targeting a specific pro-inflammatory pathway, which may lead to mild immunosuppression, are needed. JAK has been seen as a preferential target for autoimmune inflammatory diseases since its inhibition down-regulates activation of NF-κb and expression of inflammatory cytokines. In this study, the anti-flu activity of filgotinib has been identified and its combination with oseltamivir showed no adverse drug–drug interaction. The success of baricitinib, a JAK inhibitor, in COVID-19 results from its ability to decrease inflammatory cytokine level and reduce ACE2, entry receptor for SARS-COV-2, expression in airway epithelial cells [31]. Filgotinib may achieve a similar effect with its anti-inflammatory and antiviral effects in influenza and COVID-19.

In summary, we demonstrated that three immunosuppressants (MPA, 6-TG, and filgotinib) exert antiviral activity against IAV infection by targeting different phases of the IAV life cycle. MPA inhibits viral RNA replication by depletion of cellular guanosine nucleotide pool, filgotinib suppresses M1 mRNA splicing and 6-TG promotes IAV HA proteolytic degradation. Furthermore, patients infected with IAV may benefit from filgotinib treatment through simultaneously exerting anti-inflammatory and antiviral activity. Nevertheless, future clinical studies are warranted to validate our experimental findings.

## Data Availability

Data sharing is not applicable to this article as no new data were created or analyzed in this study.

## Abbreviation

IAV, Influenza A virus; MPA, Mycophenolic acid; 6-TG, 6-Thioguanine; M2, Matrix protein 2; M1, Matrix protein 1; HA, Hemagglutinin; NA, Neuraminidase; NS1, nonstructural-protein 1; ER, Endoplasmic Reticulum; UPR, Unfolded Protein Response; COVID-19, Coronavirus disease 2019; JAK, Janus kinase; m-TOR, Mammalian target of rapamycin; IMPDH, Inosine monophosphate dehydrogenase; GMP, Guanosine monophosphate; PTM, Post-translational modification.
